# Journalistic Notes

**Published:** 1890-12

**Authors:** 


### Journalistic Notes.

The *Review of Insanity and Nervous Disease*, published quarterly, has appeared with its first number. Each issue will be a complete compendium of the current literature of Neurology and Psychiatry from all languages. Articles are condensed, simplified, and, as far as possible, divested of technicality. Space will also be devoted to medico-legal literature. Every issue will represent gleanings from hundreds of journals. Special attention will be paid to literature relating to the treatment of nervous and mental diseases. Edited by J. H. McBride, M. D., Superintendent Milwaukee Sanitarium for Nervous and Mental Disease. Associate editors: Landon Carter Gray, M. D., New York City; C. K. Mills, M. D., Philadelphia; C. Eugene Riggs, M. D., St. Paul, Minn.; W. A. Jones, M. D., Minneapolis, Minn.; H. M. Bannister, M. D., Kankakee, Ill. Subscription, $2.00 per year.

---

Among our many exchanges none is held in higher esteem than the *Annals of Surgery*, published by J. H. Chambers & Co., St. Louis. This journal, under the able editorship of Drs. Pilcher and Keetley, has won a reputation second to none in the surgical world, and in the street-language of the day, "it has come to stay." It contains each month original memoirs, editorial articles, book reviews, and an index of surgical progress, comprising a synopsis of the important articles upon the various departments of surgery appearing in the recognized journals of the world. In the last ten months there have been thirty-four original articles, twenty-two editorials, upon leading topics, and 216 abstracts from the writings of the leaders in surgery. The various articles cover the entire range of surgery, and make the *Annals* a most excellent reference journal. We hope that the journal may find a large circulation, not only among surgeons but physicians as well, who will find in it much that is of use to them.

---

